# Nonsecretory Multiple Myeloma and AL Amyloidosis Presenting with Nephrotic Range Proteinuria

**DOI:** 10.1155/2015/635974

**Published:** 2015-05-21

**Authors:** Ozlem Beyler Kilic, Ali Kemal Oguz, Ihsan Ergun, Dilek Ertoy Baydar, Meltem Ayli

**Affiliations:** ^1^Department of Internal Medicine, Ufuk University School of Medicine, 06520 Ankara, Turkey; ^2^Division of Nephrology, Department of Internal Medicine, Ufuk University School of Medicine, 06520 Ankara, Turkey; ^3^Department of Pathology, Hacettepe University School of Medicine, 06100 Ankara, Turkey; ^4^Division of Hematology, Department of Internal Medicine, Ufuk University School of Medicine, 06520 Ankara, Turkey

## Abstract

Nonsecretory multiple myeloma (NSMM) is the absence of a detectable monoclonal protein in serum and urine of a multiple myeloma (MM) patient and immunoglobulin light chain (AL) amyloidosis is a significantly rare complication. A case of NSMM with AL amyloidosis and nephrotic range proteinuria is presented. Sharing clinical, therapeutic, and prognostic characteristics with MM, real challenge may be during initial diagnosis of NSMM and assessment of treatment response. In elderly patients with unexplained renal dysfunction, MM should be in the differential diagnosis and the absence of a monoclonal protein should not rule out MM but should remind us of the possibility of NSMM.

## 1. Introduction

Multiple myeloma (MM) is a hematological neoplasm of the bone marrow arising from monoclonal proliferation of plasma cells secreting a monoclonal paraprotein (M protein) which may be an immunoglobulin or one of its constituent chains [1]. Nonsecretory multiple myeloma (NSMM) is by definition the absence of a detectable M protein in the serum and the urine of an MM patient and constitutes approximately 1–5% of all patients newly diagnosed with MM [2–4].

Amyloidosis occurs with the extracellular deposition of one of a variety of abnormally folded fibrillar proteins which characteristically display a beta-pleated sheet structure. According to the Nomenclature Committee of the International Society of Amyloidosis, the clinical classification of the amyloidosis should be based on the amyloid fibril forming protein [5]. In AL amyloidosis, the deposited amyloid protein is derived from immunoglobulin light chains (i.e., lambda [*λ*] or kappa [*κ*]) originating from plasma cells [5]. One of the plasma cell dyscrasias such as MM, Waldenstrom macroglobulinemia (WM), and monoclonal gammopathy of undetermined significance (MGUS) or a B-cell non-Hodgkin's lymphoma is identified in approximately 5–15% of AL amyloidosis cases.

In the case of NSMM, the development of an AL amyloidosis is reported to be extremely rare. Herein, we present a case of NSMM complicated with AL amyloidosis resulting in nephrotic range proteinuria.

## 2. Case Presentation

A 74-year-old man was referred to our nephrology clinic on the occasion of his complaints of swollen legs and difficulty in walking. His past medical history revealed a well-controlled hypertension by valsartan/hydrochlorothiazide and doxazosin. On physical examination, he had truncal obesity, severe bilateral pretibial pitting edema, and varicose veins in his lower extremities. His routine admission laboratory tests (i.e., complete blood count, basic metabolic panel [glucose, blood urea nitrogen, creatinine, sodium, potassium, chloride, and calcium], liver panel, urinalysis, and TSH) were normal with the exceptions of low serum total protein (5.00 g/dL [6.00–8.30 g/dL]) and albumin (2.50 g/dL [3.00–5.00 g/dL]) levels together with a 300 mg/dL proteinuria on dipstick testing. While the patient's serum creatinine and eGFR (by the MDRD equation) were 0.81 mg/dL and 99 mL/min/1.73 m^2^, a 24-hour urine collection documented a proteinuria of 4.6 g/day. Simultaneously ordered serum and urine protein electrophoreses and immunofixation studies, serum-free light chain (FLC) measurements (lambda 93 mg/dL [90–210 mg/dL] and kappa 170 mg/dL [170–370 mg/dL], by nephelometry) and FLC ratio, and serum IgG, IgA, and IgM levels were all found to be normal. Antinuclear and anti-neutrophil cytoplasmic antibodies were negative and serum C3c and C4 levels were within the normal ranges.

Patient's abdominal ultrasonography documented bilaterally increased renal parenchymal echogenicities (grade 1) with renal dimensions and parenchymal thicknesses of 97 × 57 × 52/18 mm and 118 × 70 × 63/18 mm for the right and the left kidneys, respectively. A thoracic computerized tomography performed on the occasion of vague respiratory complaints revealed pleural thickening, loss of volume, and subpleural linear atelectases in the right hemithorax. As these findings were in accordance with a probable previous tuberculosis infection, a rectal mucosa biopsy was performed to search for a secondary amyloidosis. Histopathologically, no deposition of amyloid was documented in the rectal biopsy.

**Figure 1:**
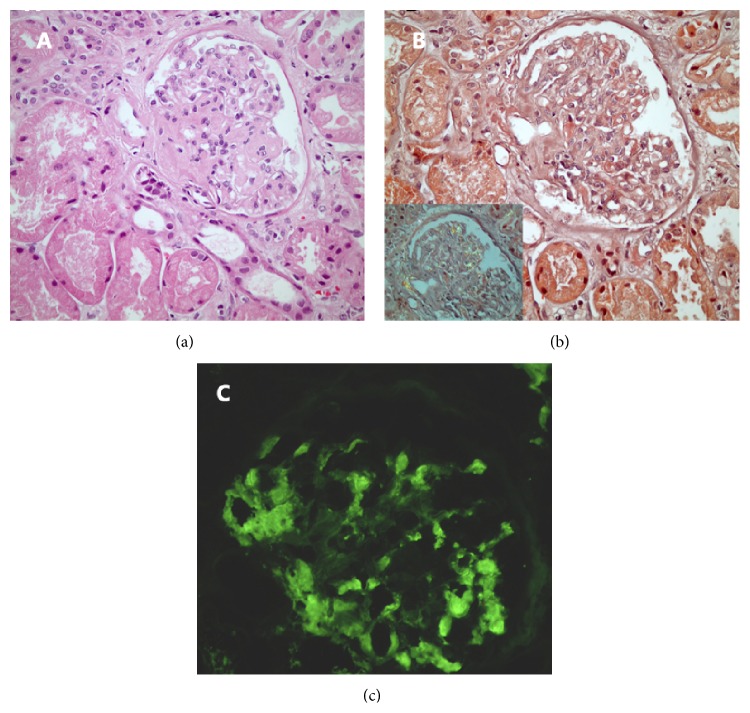
(a) Homogenous pale eosinophilic material accumulation in the glomerulus and in the hilar arteriole (H&E, ×400). (b) Positive Congo red staining in the areas of glomerular deposition (Congo red stain, ×400). Inset shows apple green birefringence given by deposits under polarized light (Congo red stain with polarized microscopy, ×200). (c) Amyloid was strongly reactive for lambda light chain on immunofluorescence microscopy (immunofluorescence, fluorescein isothiocyanate-conjugated anti-lambda antibody, ×400).

The absence of direct and clear clues about the etiology of the nephrotic range proteinuria dictated a renal biopsy which was promptly performed. Microscopic examination of the renal biopsy showed homogenous eosinophilic deposits in the glomeruli and the vessel walls which proved to be amyloid depositions with Congo red staining (Figure 1, Panels (a) and (b)). Immunofluorescence examination for lambda and kappa light chains documented a strong and a weak staining, respectively (Figure 1, Panel (c)). Consequently, the patient was diagnosed with lambda-type AL amyloidosis.

Following the diagnosis of AL amyloidosis, a bone marrow aspiration and biopsy were performed to exclude an underlying plasma cell tumor or B-cell lymphoproliferative disease. The bone marrow examination documented a uniformly appearing monotonous infiltrate of plasma cells with a percentage of 25%, which was consistent with a diagnosis of MM, in the patient's case an NSMM. Both conventional and fluorescent in situ hybridization (t[9;22], t[4;14], t[11;14], and trisomies 7 and 8 were negative) cytogenetic analyses revealed a normal karyotype (46,XY). There were lytic bone lesions of cranial and pelvic bones on conventional skeletal survey. In search of systemic involvement of AL amyloidosis, a thorough cardiac evaluation and an electroneuromyography were performed. The patient's NT-proBNP and troponin I levels were 78 pg/mL (10–110 pg/mL) and 0.04 ng/mL (≤0.04 ng/mL), respectively. On echocardiography, an interventricular septum thickness of 13 mm consistent with a mild left ventricular hypertrophy was the only abnormal finding with an ejection fraction of 64% (55–70%). Clinically, the patient was in NYHA class I. The result of the electrophysiological examination was normal.

As for chemotherapy, a combination regimen with bortezomib and dexamethasone was instituted. The response evaluation performed following the second cycle of chemotherapy documented a complete remission with a bone marrow plasma cell percentage of 4%. At the time of writing the paper, the patient was doing well with a complete remission and was waiting for a scheduled autologous stem cell transplant.

## 3. Discussion

Multiple myeloma is a hematologic neoplasm significantly more prevalent in the elderly patients [3]. The International Myeloma Working Group updated (2014) criteria for the diagnosis of MM require presence of ≥10% clonal bone marrow plasma cells and any one or more of the CRAB features (hypercalcemia, renal failure, anemia, and bone lesions) [6]. At the age of 74, having 25% monoclonal plasma cells in the bone marrow, nephrotic range proteinuria, and lytic bone lesions, the case presented herein was a typical MM patient with a nonsecretory disease. Here, it is important to note and remember that the diagnosis of MM frequently results from the diagnostic workup of elderly patients with unexplained renal dysfunction [3]. The same was true for our patient in whom a clear etiological diagnosis of nephrotic range proteinuria was made by the renal biopsy and the bone marrow examinations following the initial workup.

Conventionally, NSMM is defined as the absence of a detectable amount of M protein in the serum and urine of an MM patient [2, 4]. The previous frequency figure of NSMM among newly diagnosed MM patients was around 5%. With the widespread use of sensitive immunoassays which precisely quantify serum-free light chains, this figure is now about 3% [2, 3]. Moreover, if examined at the cellular level, great majority of NSMM plasma cells demonstrate intracellular presence of immunoglobulins or their components [7–10]. Taking this into account, some researchers group NSMM patients into two, namely, the nonproducers (about 15%; no immunoglobulin or any of its components is present in the plasma cells) and the producers (about 85%; an immunoglobulin or a component is demonstrable in the plasma cells). Consequently, it can be stated that in NSMM patients true nonproduction is unusual [8]. Although not investigated at a cellular level, the neoplastic plasma cell clone of the presented patient was presumably of the producer type as the disease resulted in a lambda-type AL amyloidosis. In addition to the state of nonproduction, several mechanisms are proposed to account for the absence of an M protein in NSMM cases. These are a decrease in M protein synthesis secondary to increased cytoplasmic immunoglobulins, accelerated degradation of abnormal intracellular immunoglobulins, impaired intracellular transport of immunoglobulins, intermittent excretion of M protein, altered secretion ability of plasma cell, reduced plasma cell membrane permeability, and rapid degradation of abnormal immunoglobulin following its secretion [8, 9].

Depending on the clinical definitions used and the diagnostic methods implemented, the incidence of renal disease in patients with MM varies between 15% and 40% [11]. The spectrum of renal diseases associating MM is considerably wide and includes many distinct clinicopathologic entities. Light chain cast nephropathy also known as myeloma kidney remains the most common form of MM related renal disease. Nonetheless, in NSMM the occurrence of light chain cast nephropathy is an unexpected situation owing to the absence of a detectable M protein, in this case a light chain in the serum and the urine of the patient [4]. The other two most common forms of M protein mediated renal disease are AL amyloidosis and monoclonal immunoglobulin deposition disease. When becoming manifest, significant proteinuria/nephrotic syndrome is present in approximately 70% of AL amyloidosis cases. The case presented had an AL amyloidosis which is documented to be very rare in patients with NSMM. Whether in series of NSMM patients or in presentations of single cases, NSMM patients with an AL amyloidosis had always been reported as individual cases and are scarce in the literature [7, 12–15]. As previously stated, the amyloid fibrils deposited in AL amyloidosis are made up of monoclonal immunoglobulin light chains secreted by the responsible plasma cell clone. Using electron microscopy, Azar et al. had also provided additional support for this fact [12].

Except for renal involvement due to light chain cast nephropathy, clinical manifestations of NSMM are consistent with MM. As such, bone marrow failure, lytic bone lesions, osteoporosis, and hypogammaglobulinemia have all been reported [4, 8, 9, 16]. In addition to a nephrotic range proteinuria, the presented case also demonstrated lytic bone lesions of cranial and pelvic bones. With respect to treatment approaches, treatment responses, and prognosis, NSMM and MM harbour shared characteristics [2, 4, 9]. A challenging point in patients with NSMM was the lack of a detectable M protein to be used during assessment of treatment responses. For this purpose, serum-free light chain measurements seem to offer a new opportunity in cases with NSMM [17].

To conclude, especially in elderly patients with unexplained renal dysfunction, MM should be included in the differential diagnosis and the absence of a detectable M protein should not rule out MM but should remind us of the possibility of an NSMM.
